# A fixable probe for visualizing flagella and plasma membranes of the African trypanosome

**DOI:** 10.1371/journal.pone.0197541

**Published:** 2018-05-16

**Authors:** Justin Wiedeman, Kojo Mensa-Wilmot

**Affiliations:** 1 Department of Cellular Biology, University of Georgia, Athens, Georgia, United States of America; 2 Center for Tropical and Emerging Global Diseases, University of Georgia, Athens, Georgia, United States of America; J. Heyrovsky Institute of Physical Chemistry, CZECH REPUBLIC

## Abstract

The protozoan *Trypanosoma brucei* sp. cause diseases in humans and animals. Studies of *T*. *brucei* cell biology have revealed unique features, such as major endocytic events being limited to a single region, and mitochondrial genome segregation mediated via basal bodies. Further understanding of trypanosome cell biology can be facilitated with super-resolution fluorescence microscopy. Lack of a plasma membrane probe for fixed trypanosomes remains a persistent problem in need of a working solution. Herein, we report protocols developed using mCLING in super-resolution structured illumination fluorescence microscopy (SR-SIM). mCLING comprehensively labels flagellar membranes, including nascent intracellular stages. To extend its usefulness for trypanosome biology we optimized mCLING in combination with organelle-specific antibodies for immunofluorescence of basal bodies or mitochondria. Then in work with live trypanosomes, we demonstrated internalization of mCLING into endocytic stations that overlap with LysoTracker in acidic organelles. Greater detail of the intracellular location of mCLING was obtained with SR-SIM after pulsing trypanosomes with the probe, and allowing continuous uptake of fluorescent concanavalin A (ConA) destined for lysosomes. In most cases, ConA and mCLING vesicles were juxtaposed but not coincident. A video of the complete image stack at the 15 min time point shows zones of mCLING staining surrounding patches of ConA, consistent with persistence of mCLING in membranes of compartments that contain luminal ConA. In summary, these studies establish mCLING as a versatile trypanosome membrane probe compatible with super-resolution microscopy that can be used for detailed analysis of flagellar membrane biogenesis. In addition, mCLING can be used for immunofluorescence in fixed, permeabilized trypanosomes. Its robust staining of the plasma membrane eliminates a need to overlay transmitted light images on fluorescence pictures obtained from widefield, confocal, or super-resolution microscopy.

## Introduction

*Trypanosoma brucei* is a protozoan that causes Human African Trypanosomiasis and *nagana* in cattle (reviewed in [1–2]). *T*. *brucei* has been the subject of many studies to understand unique aspects of its biology (*e*.*g*. antigenic variation, RNA editing, trans-splicing, and catenated mitochondrial DNA). Fluorescence microscopy is frequently used to visualize cells, localize trypanosome proteins [3], and detect small molecule drugs intracellularly [4]. Resolution of the technique is constrained by the diffraction limit of visible light, as it cannot distinguish fluorescent objects within ≈200 nm lateral to each other (reviewed in [5]). Super-resolution fluorescence microscopy techniques, such as structured illumination and stimulated emission depletion (STED) enable resolution of objects within 200 nm lateral to each other [5–6], and have been used to study *T*. *brucei* [7,8]. For a cell as small as a trypanosome (≈2 μm wide, ≈25 μm long), super-resolution microscopy offers notable advantages over standard fluorescence microscopy.

One vexing limitation of data acquired by super-resolution microscopy of *T*. *brucei* is the inability to demarcate the periphery of fixed cells, which is crucial for orientation of fluorescent organelles and intracellular macromolecular structures. Outlining the limiting membrane of the trypanosome in regular fluorescent microscopy has traditionally been accomplished using one method. Transmitted light images (*e*.*g*. differential interference contrast or phase contrast) are captured and superimposed on the fluorescence image. Unfortunately, microscopes that integrate high-quality transmitted light capabilities with super-resolution fluorescence are not commercially available. One solution to this limitation of super-resolution microscopy is to stain the plasma membrane with a fluorescent probe. However, no “fixable”, fluorescent probe is available for routine use in super-resolution microscopy of bloodstream form trypanosomes.

mCLING is a lipopeptide conjugate dye developed to track neuronal membranes using super-resolution microscopy [9]. In this study, we report protocols that use mCLING to label trypanosome flagella, plasma membrane, and endosomes. Further, we present optimized work-flows for using mCLING in combination with antibodies specific for intracellular organelles, thereby expanding the versatility of mCLING for super-resolution microscopy in bloodstream form *T*. *brucei*.

## Materials and methods

### Trypanosome culture

Bloodstream form *T*. *brucei* Lister 427 was cultured in HMI-9 media [10] in log-phase growth (≤10^6^ cells/mL) [3].

### mCLING labeling of bloodstream form *T*. *brucei*

Trypanosomes (2x10^6^) were harvested, pelleted by centrifugation (3 min, 3000 xg), and washed in cold phosphate-buffered saline (PBS) supplemented with 10 mM glucose (PBS-G). Trypanosomes were centrifuged again and re-suspended in 100 μL cold PBS-G. mCLING-488 (Catalog number 710006AT3(SY), Synaptic Systems Gmbh, Goettingen, Germany) was added to a final concentration of 1 μM, and cells were gently mixed with a pipette. Cells were incubated on ice in the dark for 15 minutes.

### mCLING and DAPI co-staining

Trypanosomes were labeled with mCLING (described above), then fixed by adding 450 μL ice-cold, freshly mixed 4% paraformaldehyde (PFA) in PBS (Affymetrix, Santa Clara, CA) containing 0.05% glutaraldehyde, and incubated for 1 minute at room temperature (RT). Cells were pelleted (3 min, 3000 xg, RT) and all but 10 μL of the fixative was aspirated. The trypanosome pellet was re-suspended in the remaining fixative and dotted onto parafilm. Glass cover slips coated with poly-L-lysine (Sigma-Aldrich, St. Louis, MO) were placed on top of the cells, and incubated at room temperature in the dark for 10 minutes to adhere trypanosomes to the cover slip. Cover slips were transferred to a 24-well plate and gently washed with PBS. Five hundred microliters of 100 mM glycine/100mM ammonium chloride in PBS was added to each well to quench unreacted amines, and cover slips were incubated for 10 minutes in the dark at room temperature. The quenching solution was aspirated and the cover slips were rinsed in PBS. Cover slips were briefly immersed in de-ionized water, gently dried with a Kim-wipe, and mounted onto microscope slides with VectaShield Mounting Medium (Vector Laboratories, Burlingame, CA) containing 4’,6-diamidino-2-phenylindole (DAPI). Images of cells were acquired with an Elyra S1 SR-SIM microscope (Zeiss, Jena, Germany). Maximum intensity projections of the acquired 3D image stacks, and brightness and contrast adjustments were performed in Fiji [11].

### Immunofluorescence assays

Trypanosomes were labeled with mCLING and fixed as described above. Cover slips were then immersed in 50 mL freshly prepared “antigen retrieval” buffer (100 mM Tris with 5% w/v urea, pH 9.5) [12] and incubated at 95^°^C for 1 min (for cells to be probed with YL1/2) or 5 min (for cells to be probed with anti-TAO mAb). Cover slips were transferred to a 24-well plate, rinsed with PBS, and permeabilized with 500 μL 0.15% Triton X-100 (Thermo Fisher) in 1% bovine serum albumin (BSA) (Sigma-Aldrich) in PBS for 45 minutes. The permeabilization buffer was aspirated, and cover slips were incubated with either YL1/2 antibody (1:1000) (EMD Millipore, Billerica, MA) or anti-trypanosome alternative oxidase (TAO) antibody (1:50) (gift from Dr. Minu Chaudhuri, Meharry Medical College, Nashville, TN) (each diluted in PBS containing 0.15% Triton X-100 and 1% BSA) for 40 minutes. Cover slips were rinsed 3 times with PBS, and then incubated with secondary antibody (1:3000 AlexaFluor-594 goat-anti-rat for YL1/2 or 1:3000 AlexaFluor-594 goat-anti-rabbit for anti-TAO (Molecular Probes, Eugene, OR)) for 40 minutes. Cover slips were washed 3 times in PBS, briefly immersed in de-ionized water, gently dried with a Kim-wipe, and mounted on a microscope slide with VectaShield Mounting Medium containing DAPI. An Elyra S1 SR-SIM microscope was used to capture images of trypanosomes. Maximum intensity projections of the acquired 3D image stacks, and brightness and contrast adjustments were performed in Fiji [11].

### Endocytosis assays

Trypanosomes (6x10^6^) were harvested, pelleted by centrifugation (3 min, 3000 xg), and washed in cold PBS-G. Cells were centrifuged again and re-suspended in 300 μL cold PBS-G. mCLING-488 and Concanavalin A-AlexaFluor-594 conjugate (Life Technologies, Carlsbad, CA) were added to final concentrations of 1 μM and 50 μg/mL, respectively, and cells were gently mixed with a pipette. Cells were incubated on ice in the dark for 15 minutes. One hundred microliters of trypanosomes were transferred to a separate centrifuge tube and fixed by adding 450 μL ice-cold 4% PFA containing 0.05% glutaraldehyde, on ice. For the remaining trypanosomes, exogenous mCLING and ConA-594 were removed by centrifuging at 4^°^C (3 min, 3,000 xg). The cell pellet was re-suspended in 200 μL ice-cold PBS-G containing 50 μg/mL ConA-594, and divided into two equal aliquots, which were incubated in the dark (37^°^C with 5% CO_2_), for 5 or 15 minutes. Immediately following incubation, samples were placed on ice and fixed exactly as described in the section on mCLING and DAPI co-staining.

### mCLING and LysoTracker imaging flow cytometry of trypanosomes

Trypanosomes (2x10^6^) were washed in cold PBS-G and re-suspended in 50 μL cold PBS-G and kept on ice until analysis. LysoTracker Deep Red (Life Technologies, Carlsbad, CA) was added to a final concentration of 50 nM in 0.2% DMSO, and mCLING-488 was then added to a final concentration of 250 nM. Samples were mixed by gentle agitation and immediately loaded into an ImageStream X Mark II (Amnis, Seattle, WA) imaging flow cytometer at room temperature. (According to communication with Amnis product engineers, the internal temperature of the ImageStream is approximately 2^°^C warmer than ambient temperature (room temperature ≈25^°^C)). mCLING-488 was excited by a 488 nm laser (35mW) and emission was detected using a 533/55 filter. LysoTracker Deep Red was excited by a 642 nm laser (25mW) and emission was detected using a 702/85 nm filter. Images were acquired for at least 5 minutes. Data analysis was performed in IDEAS software, version 6.2.187.0 (Amnis, Seattle, WA). Colocalization of mCLING and LysoTracker was assessed using the “Colocalization Wizard”. The “MC” mask in IDEAS was used to define the image space occupied by the trypanosome used for the analysis. Example images illustrating colocalization scores for colocalization were adjusted to similar brightness and contrast levels in IDEAS. Four-fold less mCLING-488 was used for imaging flow cytometry than for SR-SIM. Experiments with SR-SIM required a higher concentration of mCLING to enable visualization of the membrane present in a single focal plane. The imaging flow cytometer took single images of the entire cell (~2–4 um thick), illuminating all theoretical focal planes at once. In this scenario, emission from 1 μM mCLING-488 was oversaturating the detector. Thus the concentration of mCLING 488 was decreased from 1 μM to 250 nM.

### Statistical analysis of imaging flow cytometry data

mCLING/LysoTracker colocalization scores were computed for each imaged trypanosome. Trypanosomes that were imaged within 2.5 second of the selected time points (0, 1.25, 2.5, 5, and 15 minutes) were analyzed as follows. Colocalization scores for each time point were pooled from 3 biological replicates and analyzed together. N>30 for each replicate at each time point. Total number of trypanosomes at each time point ≥ 133. Statistical testing was performed in Prism, version 7.03, (GraphPad Software, La Jolla, CA). Significance between means of colocalization scores was tested using one-way ANOVA (Brown-Forsythe F (4,827) = 9.941, p < 0.0001), with subsequent Tukey’s analysis to determine statistical significance between specific pairs of means. A P-value <0.05 was considered statistically significant.

## Results

### mCLING labels the flagellum and plasma membranes of bloodstream form *T*. *brucei*

Our primary objective was to identify a fluorescent membrane probe that could be used to outline a fixed trypanosome cell for use in super-resolution microscopy. Transmitted light microscopy has traditionally been used to outline trypanosomes, but commercial super-resolution fluorescence microscopes capable of transmitted light imaging are unavailable. To this end, we developed a protocol for robust mCLING labeling of *T*. *brucei*.

**Fig 1:**
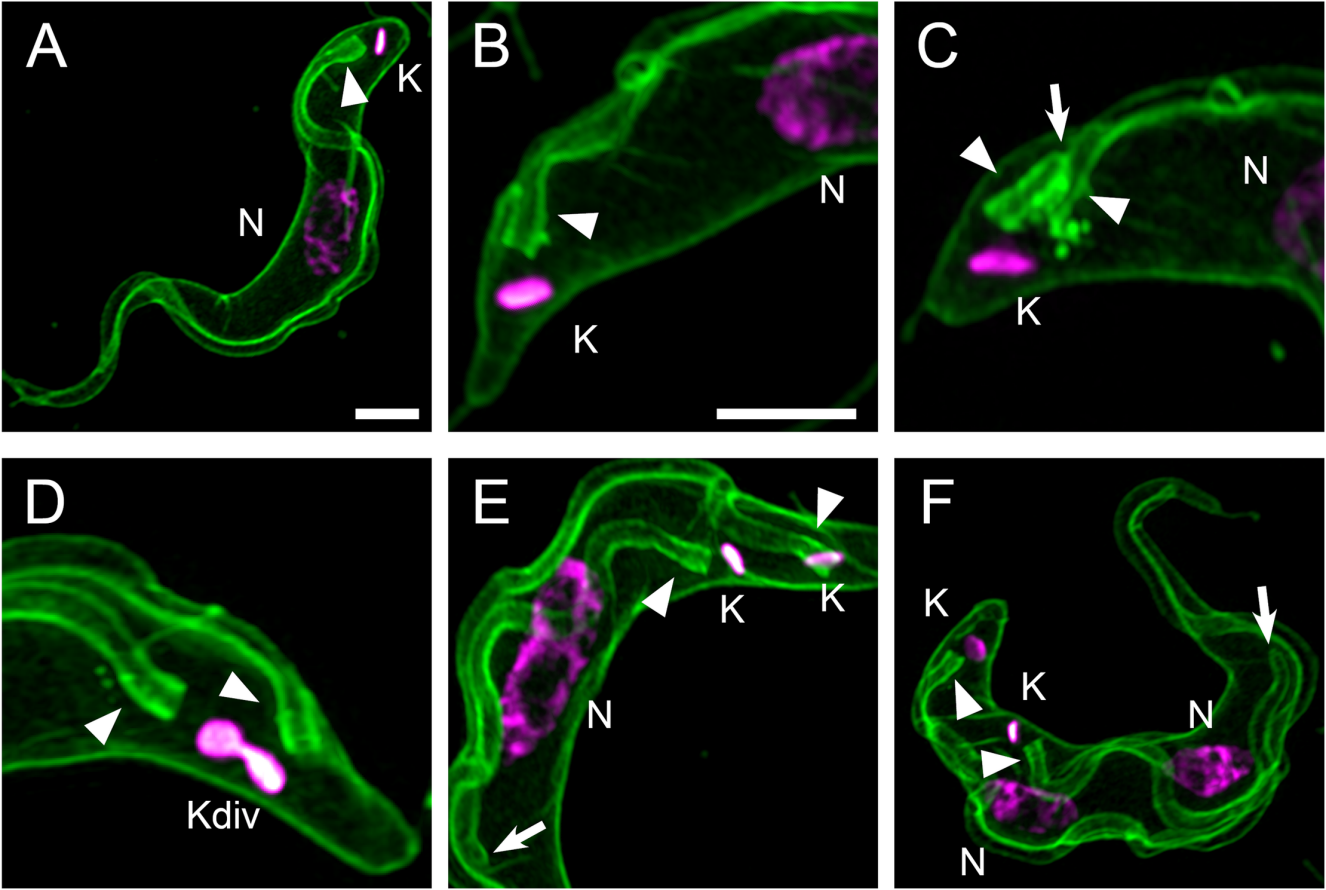
mCLING labels the plasma and flagellar membranes. Trypanosomes were incubated with mCLING-488 for 15 min on ice, fixed, and the kinetoplast (K) and nuclear (N) DNA was stained with DAPI. Panels presented are maximum intensity projections of 3D SR-SIM images. The “magenta hot” look-up table in Fiji (that colors areas of intense signal white) was applied to the DAPI channel. (A) Plasma and flagellar membrane staining of a whole trypanosome. (B) Plasma and flagellar membranes of a 1K1N trypanosome. (C) Plasma and flagellar membranes of a trypanosome that has two flagella, labeled with arrowheads. (D) Further growth of a nascent flagellum and apparent division of an elongated kinetoplast (Ke). (E) Elongation of the nucleus (compare to panel A) during mitosis, following kinetoplast division. Arrow points to the anterior tip of a growing flagellum. (F) Post-mitotic cell with two kinetoplasts and two nuclei. Arrow points to the tip of a growing flagellum. Arrowheads indicate the posterior bases of flagella. K = kinetoplast. Kdiv = dividing kinetoplast. N = nucleus. Scale bar = 2 μm.

3D Super-resolution structured illumination fluorescence microscopy (SR-SIM) using mCLING revealed detailed staining of the flagellar membrane. The flagellar membrane originated from the posterior end of the trypanosome near the kinetoplast (mitochondrial nucleoid) (Fig 1A and 1B), and it traversed the length of the cell (Fig 1H). An area of intense mCLING staining consistent with its assignment as the flagellar pocket [13] is detected at the base of each flagellum (Fig 1B).

Formation of a nascent flagellum is detected by mCLING staining. Prior to division of a kinetoplast [3,14], a new flagellum protrudes from a newly-formed flagellar pocket alongside the old flagellum [15] (Fig 1C) and continues to elongate through the period when the kinetoplast divides (Fig 1D–1F). The *T*. *brucei* flagellum has previously been visualized by immunofluorescence of axonemal or paraflagellar rod proteins (such as PFR [16]). To our knowledge this is the first time the entire flagellar membrane in a fixed bloodstream form trypanosome has been visualized with fluorescence microscopy.

### Coupling of antibody detection of proteins with mCLING in fluorescence microscopy

**Fig 2:**
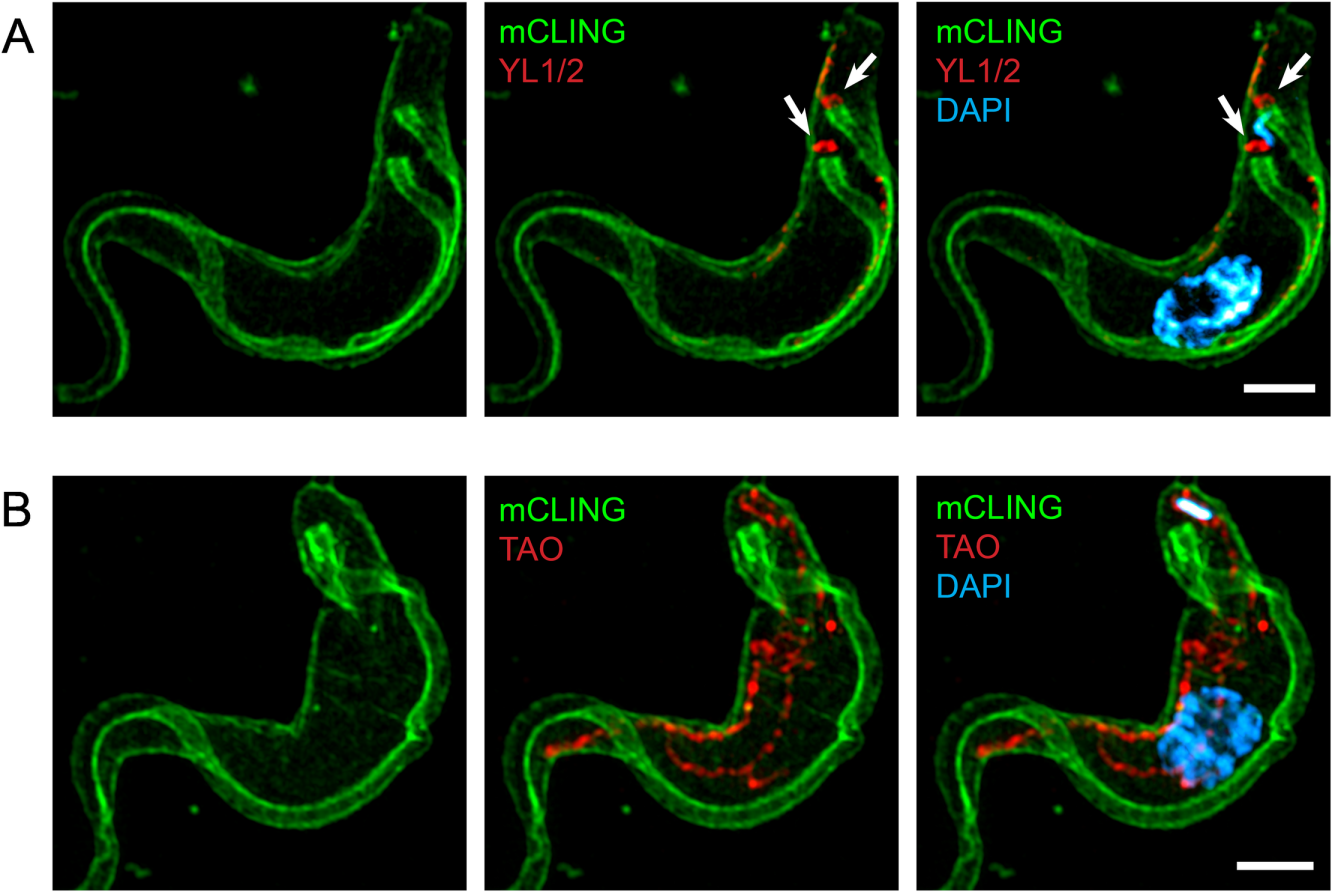
Double-labeling of trypanosomes with antibodies and mCLING. Trypanosomes were incubated with mCLING-488 on ice, then fixed and subjected to “antigen retrieval” before incubation with primary and fluorescent secondary antibodies. The kinetoplast and nucleus were stained with DAPI. Panels presented are maximum intensity projections of 3D SR-SIM images. (A): The trypanosome basal body protein TbRP2 was labeled with YL1/2 primary antibody and AlexaFluor-594 secondary antibody. (B): Trypanosome mitochondrial alternative oxidase (TAO) was labeled with anti-TAO primary antibody and AlexaFluor-594 secondary antibody. Scale bar = 2 μm.

We recognized that usefulness of mCLING in trypanosome biology could be expanded if investigators were able to employ the probe in immuno-localization experiments. Therefore, we attempted to optimize mCLING for use in combination with protein-specific antibodies. For this purpose, we incubated trypanosomes for 15 minutes on ice with mCLING and then fixed the cells with paraformaldehyde (PFA) and glutaraldehyde. Trypanosomes were then subjected to “antigen retrieval” to reverse epitope masking caused by glutaraldehyde fixation (data not shown) [12,17,18]. Cells were permeabilized and probed with either YL1/2 antibody to detect TbRP2 at basal bodies [19], or an antibody against a mitochondrial trypanosome alternative oxidase (TAO) [20]. Images of trypanosomes captured with 3D SR-SIM revealed mCLING staining of the plasma and flagellar membranes simultaneous with antibody detection of basal bodies when YL1/2 was used (Fig 2A). In the case of anti-TAO/mCLING staining, the mitochondrion was revealed as a tubular network throughout the length of the trypanosome (Fig 2B) [21], while mCLING labeled the plasma membrane. We conclude that mCLING can be used to label the plasma membrane in fixed trypanosomes for immunofluorescence studies to detect intracellular organelles.

### After uptake, mCLING is detected near acidic compartments

**Fig 3:**
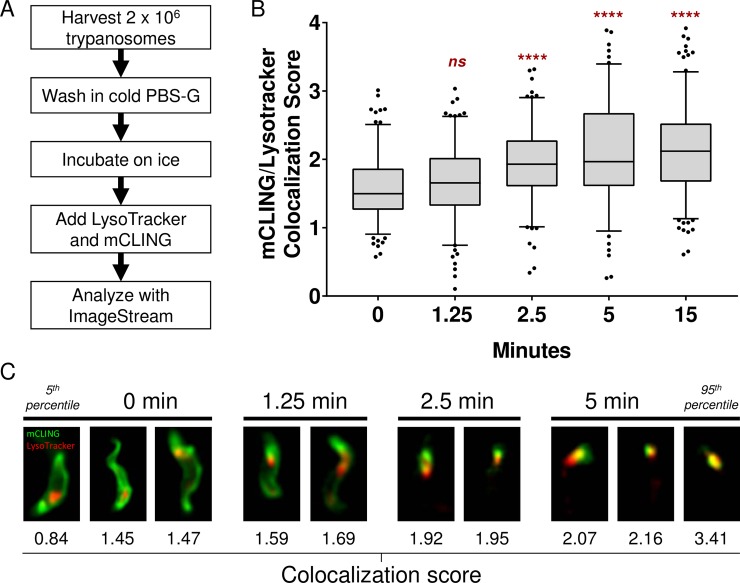
A fraction of mCLING colocalizes with acidified intracellular compartments. Uptake of mCLING and LysoTracker was observed in real-time with imaging flow cytometry. Colocalization of mCLING and LysoTracker was assessed using IDEAS software Colocalization Wizard. (A) Flow chart of the experimental procedure. (B) Box and whisker plot of the colocalization score over time. Data points collected within 2.5 seconds of the indicated time points from 3 biological replicates were combined and graphed. N>30 for each replicate at each time point. N≥133 for each time point. Statistical significance between means (1.578, 1.673, 1.935, 2.106, and 2.124, respectively) was determined using one-way ANOVA (p < 0.0001). Annotations in red above each box and whisker plot represent the results of Tukey’s test between time 0 and the respective time point (**** = p <0.0001, ns = not significant). Whiskers represent 5^th^ and 95^th^ percentiles. (C) Example images of trypanosomes with colocalization scores close to the medians (1.498, 1.657, 1.93, and 1.967, respectively) acquired during the collection periods for the indicated time points. Images representing 5^th^ and 95^th^ percentiles of the 0 min and 5 min time points, respectively, are presented.

Movement of mCLING from the plasma membrane into the trypanosome was tracked with LysoTracker, which accumulates in acidified endosomes and the lysosome [22]. In our protocol, cultured trypanosomes were washed with cold PBS-G and kept on ice. LysoTracker and mCLING were added to the live trypanosomes, which were immediately loaded into an imaging flow cytometer (Fig 3A). Acquisition of images on the cytometer began approximately 60 seconds (64.3, +/- 2.5 seconds, data not shown) after cells were loaded, and data was captured for at least 5 minutes. The data were analyzed with IDEAS software.

At early time-points (0–1.25 min), mCLING preferentially labeled the plasma membrane whereas LysoTracker was intracellular (Fig 3C). At 2.5 min and beyond, the mCLING signal on the plasma membrane of the trypanosome faded while pockets of the probe accumulated near LysoTracker-positive compartments (Fig 3B). The degree of colocalization between mCLING and LysoTracker was determined using the Colocalization Wizard in IDEAS. From this data set, colocalization scores for trypanosomes imaged over a 2.5-second span at different time points were graphed (Fig 3C) and analyzed with Prism. There was a statistically significant difference between colocalization scores of groups as determined by one-way ANOVA (p < 0.0001). Subsequent Tukey’s analysis revealed the difference in mean colocalization scores was not statistically significant (p = 0.5957) between 0 minutes (mean = 1.578) and 1.25 minutes (mean = 1.673) for mCLING and LysoTracker. However, after 2.5 min, the difference in the mean colocalization scores for mCLING/LysoTracker was statistically significant (p < 0.0001) between 0 and 2.5 minutes (mean = 1.935), 0 and 5 minutes (mean = 2.106), and 0 and 15 minutes (mean = 2.124). At the 5 min time point, extensive juxtaposition of mCLING and Lysotracker was detected, whereas the probe was not detected on the plasma membrane to the same extent as it was within the first minute (Fig 3B). From these data, we conclude that mCLING is endocytosed in unfixed trypanosomes and that after 5 min its intracellular location overlaps with acidic compartments in the trypanosome.

### *T*. *brucei* endocytosis of mCLING at 37^°^C

**Fig 4:**
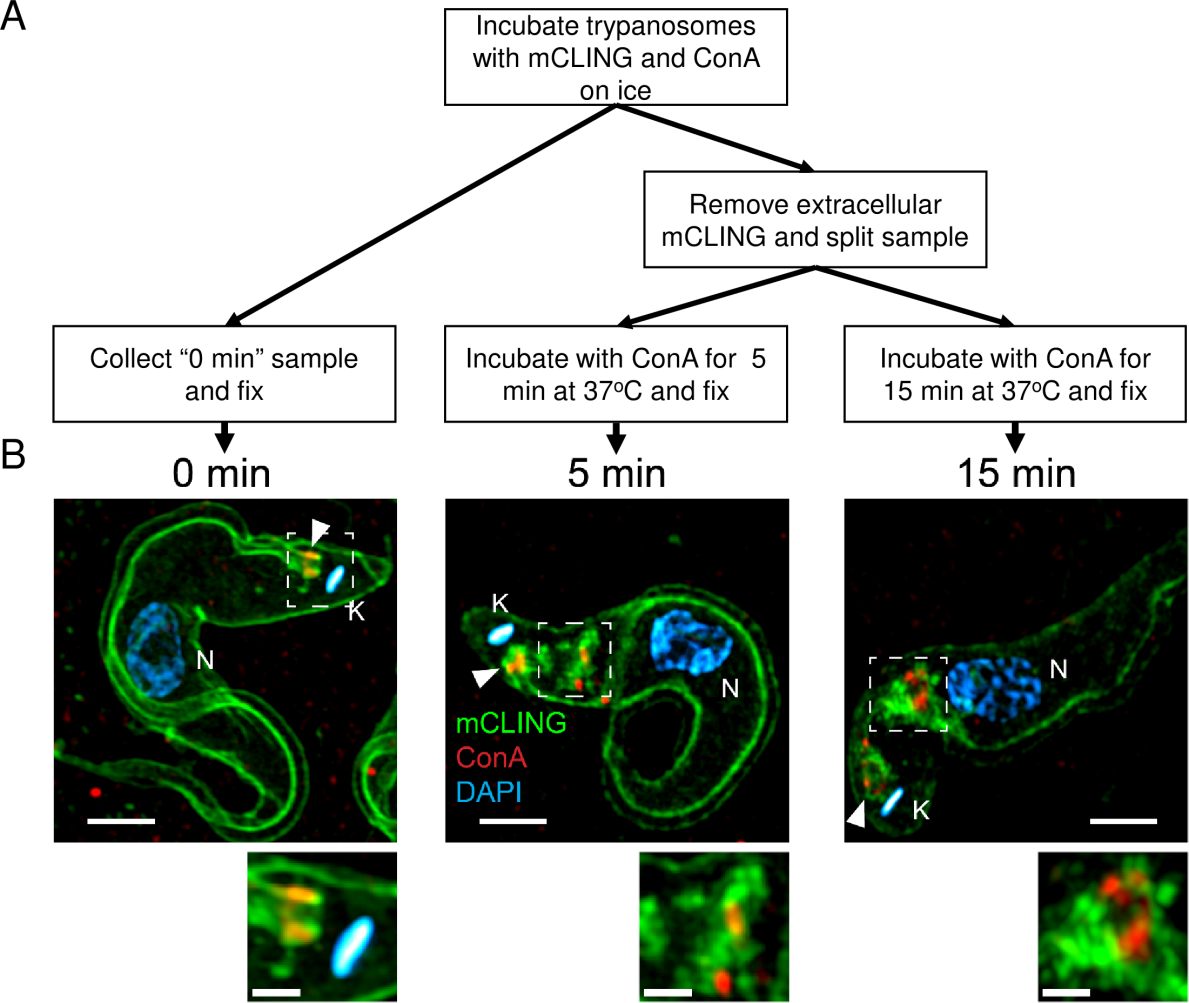
Labeling of endosomes with ConA and mCLING. Trypanosomes were incubated with mCLING-488 and ConA-594 for 15 minutes on ice, then exogenous mCLING was removed. Cells were incubated at 37^°^C with ConA-594 for 5 or 15 minutes and fixed. DNA in the kinetoplast and nucleus was stained with DAPI. (A) Simplified flow chart of the experimental procedure. (B) Maximum intensity projections of 3D SR-SIM images. 0 min represents the “No uptake” control (kept on ice); 5 min and15 min images include internalized mCLING-positive endosomes. Boxes (in 5 min and 15 min samples) indicate areas of the trypanosome containing endocytosed mCLING and ConA. ConA is observed at the base of the flagellum in the 0 min and 5 min samples. Small image panels below the larger images show the area enclosed by the box, at a higher magnification. K = kinetoplast. N = nucleus. Arrowheads indicate the posterior bases of flagella. Scale bar in large panels = 2 μm. Scale bare in small panels = 1 μm.

We investigated whether mCLING could be used to track endocytosed plasma membrane in bloodstream form *T*. *brucei*. For this goal, we monitored clearing of mCLING from the plasma membrane and subsequent accumulation in intracellular vesicles in trypanosomes incubated at 37^°^C. Bloodstream form trypanosomes are typically cultured at 37^°^C, as this is the approximate body temperature of vertebrates infected with *T*. *brucei*. The lectin concanavalin A (ConA) was used to label some endocytic vesicles that terminate in lysosomes [23,24]. Trypanosomes were incubated with mCLING and ConA on ice for 15 minutes, after which excess mCLING was washed off. Trypanosomes were re-suspended in buffer containing ConA and incubated at 37^°^C (Fig 4A). Images were acquired using 3D SR-SIM and processed with Fiji [11].

Labeling of mCLING at the plasma membrane decreases over time when trypanosomes are incubated at 37^°^C without exogenous mCLING (Fig 4). Trypanosomes incubated only on ice with mCLING had strong signal on the plasma membrane without significant internalization of the probe (Fig 4B, 0 min). After 5 minutes at 37^°^C, the plasma membrane mCLING signal decreased whereas new zones of intense mCLING staining appeared inside the trypanosomes between the flagellar pocket and nucleus (Fig 4B). After 15 minutes at 37^°^C, the intracellular mCLING signal remained intense while the plasma membrane signal appeared to diminish (Fig 4B).

ConA initially accumulated at the base of the flagellum (Fig 4B, 0 min) when trypanosomes were incubated on ice [23]. After 5 minutes of incubation in buffer containing ConA at 37^°^C, the lectin was observed at the base of the flagellum and intracellularly between the kinetoplast and nucleus (Fig 4B 5 min). Some internalized ConA co-localized with a fraction of mCLING, but most mCLING did not co-localize with ConA. After 15 minutes at 37^°^C, ConA was found at both the flagellar pocket (due to continuous uptake from the medium) and in an endocytic station (most likely the lysosome [24]) closer to the nucleus than the kinetoplast (Fig 4B, 15 min). In most cases, ConA and mCLING vesicles were juxtaposed but not coincident, implying that ConA and mCLING have different endocytic fates and/or recycling. Alternatively, ConA is in the lumen of a multivesicular body whereas mCLING remains membrane-associated.

## Discussion

Fluorescence microscopy is an important tool for trypanosome cell biology [22], and recent technical advancements have enabled the visualization of objects smaller than the resolution limit imposed by diffraction of light. In trypanosome biology, few “fixable”, fluorescent, non-reactive probes for labeling the plasma membrane of bloodstream form *T*. *brucei* are available [25], and these are not known to be compatible in common super-resolution techniques that have specific fluorophore requirements [9]. We have attempted to fill this gap in fluorescence microscopy of bloodstream form trypanosomes. Using 3D SR-SIM, we demonstrate that the lipopeptide probe mCLING labels plasma and flagellar membranes, and endocytic stations. Further, mCLING is fixable and can be used for immunofluorescence microscopy.

mCLING vividly labels flagellar and plasma membranes in *T*. *brucei* (Fig 1). 3D SR-SIM imaging of mCLING-labeled trypanosomes revealed details of the flagellar membrane only clearly observed previously with electron microscopy [26]. For example, membranes of the nascent flagellum and flagellar pocket are detected with mCLING before the flagellum protrudes from the cell body (Fig 1C, S2 and S3 Videos). The ease with which the flagella membranes are labeled by mCLING suggest that it could find use in analysis of mutants of flagellar biogenesis [27–28], specifically in identifying those that prevent new membrane recruitment to the flagellum. In addition to providing a clear view of the trypanosome plasma membrane, mCLING enables super-resolution 3D reconstruction of the trypanosome cell body (S1 Video). To the best of our knowledge, this is the first report of whole-trypanosome 3D reconstruction using super-resolution fluorescence microscopy.

In recognition of mCLING’s versatility as a membrane probe, we adapted it for trypanosome immunofluorescence assays (IFA). In our hands, classic lipophilic membrane dyes are poorly retained in the plasma membrane after fixation of bloodstream form *T*. *brucei*, even when using “fixable” derivatives of such dyes (e.g. FM4-64FX (data not shown)). The absence from the literature of images of fixed, plasma membrane-stained bloodstream form *T*. *brucei* suggests that limitation in the use of strictly lipophilic dyes for trypanosome plasma membrane staining is not easily overcome: mCLING solves this problem. Using mCLING, we achieved robust staining of the plasma and flagellar membranes in fixed cells labeled with antibodies for a basal body protein or mitochondrial protein (Fig 2). This procedure makes obsolete a need to overlay a transmitted light image of *T*. *brucei* (or hand-draw the outline of cells) on fluorescence images obtained in super-resolution, widefield, and confocal microscopy. In addition, 3D imaging of mCLING in an IFA can reveal the location of organelles in the vertical dimension relative to the trypanosome cell body, a technique that is not possible with an image overlay approach.

Imaging flow cytometry of live, unfixed, bloodstream form trypanosomes demonstrated that mCLING is taken up via endocytosis and that it is directed proximal to acidified vesicles (Fig 3C). The degree of colocaliztion of mCLING with LysoTracker increases for the first 5 minutes (Fig 3B), then remains constant until at least the 15 minute time point. mCLING’s rapid internalization is similar to that of FM4-64 [22]. While imaging flow cytometry rapidly samples large numbers of trypanosomes, the image resolution along the z-axis is constrained, limiting the ability to differentiate between mCLING on the surface of an acidic compartment and mCLING contained in the lumen of an acidic compartment.

The precise location of mCLING in the endocytic system was determined with SR-SIM. In an experiment combining a “pulse” of mCLING with continuous ConA (which is known to localize to the lysosome [24]) uptake at 37^°^C, ConA and mCLING signals gradually diverge as incubation time increases (5 min vs 15 min) (Fig 4B), suggesting that mCLING and ConA have unique endocytic fates. ConA signal at the base of the flagellum decreases between the 5 and 15 minute time points, likely due to the gradual clearance of ConA that accumulated during the initial 15 minute incubation on ice (Fig 4A). A video of the complete image stack for Fig 4B, 15 min sample, (S4 Video), shows areas of mCLING staining that surround segments of ConA, consistent with the concept that mCLING is integrated into the membrane of endocytic compartments that contain ConA in their lumen. Imaging flow cytometry (Fig 3B) demonstrates that mCLING is taken up and transported to regions proximal to acidic compartments by 5 minutes. ConA does not appear to be transported intracellularly at the same rate as mCLING. A recent report did not report significant colocalization between ConA and p67 (a lysosomal marker) until 10–20 minutes after uptake [29]. Thus it is likely that at the 5 minute time point ConA cargo is still in mCLING-labeled vesicles destined for the lysosome. By the 15 minute time point, ConA cargo has likely been deposited in the lysosome while the mCLING-labeled membrane is likely subject to recycling [30].

The modular structure of mCLING (i.e. a fluorophore conjugated to a lipopeptide) confers some desirable properties that improve its versatility. The probe is an octapeptide containing one cysteine and seven lysines, one of which is linked to a palmitoyl group in an amide linkage through an N-hydroxysuccinimide ester (NHS ester) reaction [9]. The fluorophore is attached to the peptide by maleimide chemistry to the cysteine, resulting in a stable thioether bond [9]. First, mCLING can theoretically be conjugated to any maleimide-containing fluorophore. Hence, many other fluorophores compatible with multiple super-resolution techniques can be used with mCLING [31,32]. FM-series dyes, by contrast, are not known to be compatible with common super-resolution microscopy techniques that require specific fluorophore properties. Second, fluorophores of various excitation and emission properties can be used with mCLING. From this perspective, mCLING is an important addition to tools available for fluorescence microscopy of *T*. *brucei*, including widefield, confocal, and super-resolution techniques.

In summary, we have established mCLING as a multi-faceted membrane probe for super-resolution imaging of bloodstream form *T*. *brucei*. mCLING robustly labels plasma and flagellar membranes (Fig 1), and can be used in immunofluorescence assays (Fig 2). mCLING can be used to track endocytic vesicles (Figs 3 and 4). Our work with mCLING-488 was performed using 3D SR-SIM, which provides a ~2-fold increase in resolution over classic fluorescence microscopy (reviewed in [5]). However, mCLING with other fluorophores has been used in other super-resolution techniques, including STED, PALM, and STORM [9,31,32], that provide greater increases in resolving power. We anticipate that using mCLING with these other super-resolution techniques will reveal even finer details of trypanosome membrane and organelle biology.

## Supporting information

S1 Video 3D-rendering of SR-SIM images. Trypanosomes were labeled with mCLING and DAPI as described in *Materials and Methods*. A “z-stack” of images was sharpened, adjusted for brightness and contrast, and 3D-rendered using maximum intensity projection, in Zen 2012 Blue Edition (version 1.1.2.0) (Carl Zeiss Microscopy, GmbH). Green = mCLING, Magenta = DAPI.
(MP4)

S2 Video 3D reconstruction of data presented in Fig 1C. The lumens of the mature and nascent flagella are colored yellow and red, respectively. The lumen of each flagellum was traced using the “Simple Neurite Tracer” (SNT) plugin (version 3.1.6) (Longair MH, Baker DA, Armstrong JD. Simple Neurite Tracer: open source software for reconstruction, visualization and analysis of neuronal processes. Bioinformatics. 2011;27: 2453–2454. doi:10.1093/bioinformatics/btr390) found in Fiji. (A) A 3D surface rendering of the area of the lumen was created using SNT and combined with the original SR-SIM image file. To reduce pixel “noise”, the original SR-SIM image was processed using the “Median 3D” command in Fiji prior to combining with the surface-rendered flagellar lumens.
(MP4)

S3 Video 3D projection of data presented in Fig 1C. The lumens of the mature and nascent flagella are colored yellow and red, respectively. The surface-rendered flagellar lumens were created using SNT and combined with the original SR-SIM image stack. The stack was rendered using the “3D project” command in Fiji.
(MP4)

S4 Video Fly-through of image planes from Fig 4 (15 min) SR-SIM image stack. Images were adjusted for brightness and contrast in Fiji. Green = mCLING. Red = ConA. Cyan = DAPI.
(MP4)
